# Optimization of the transseptal procedural workflow using a novel integrated dilator and needle during a cryoballoon procedure

**DOI:** 10.1016/j.hrcr.2021.11.001

**Published:** 2021-11-09

**Authors:** Sing-Chien Yap, Tamas Szili-Torok

**Affiliations:** Department of Cardiology, Erasmus MC, University Medical Center Rotterdam, Rotterdam, the Netherlands

**Keywords:** AcQCross Qx, AcQGuide Max, Atrial fibrillation, Cryoballoon ablation, Pulmonary vein isolation, Transseptal access, Transseptal sheath

## Introduction

**Figure 1:**
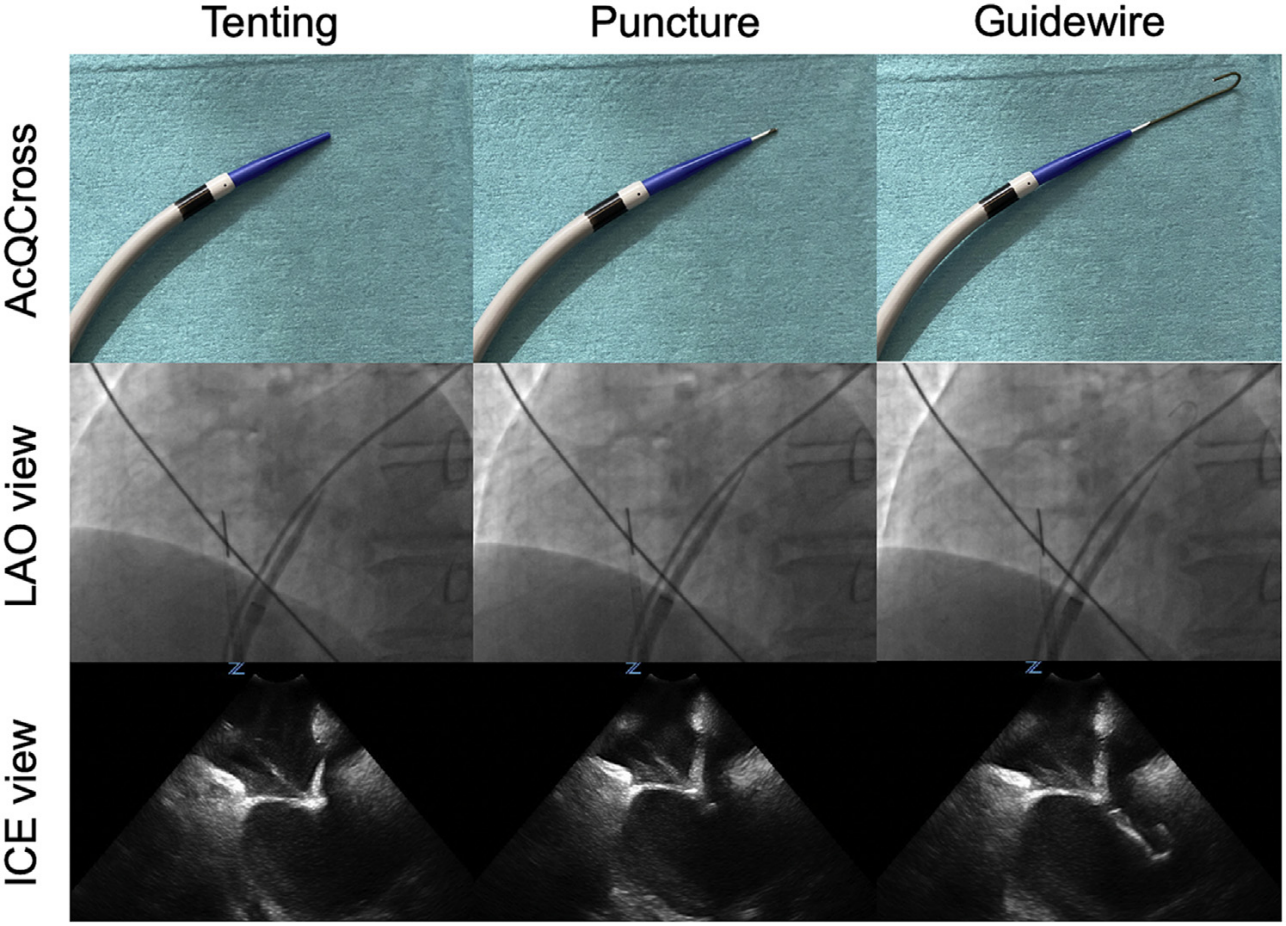
Imaging of the AcQCross dilator/needle system (Acutus Medical, Carlsbad, CA) during the different steps of a transseptal puncture. After visualization of tenting of the fossa ovalis during intracardiac ultrasound imaging, the needle is advanced and the retained 0.032” guidewire is advanced to the left superior pulmonary vein. ICE = intracardiac echocardiography; LAO = left anterior oblique.

Cryoballoon ablation of the pulmonary veins (PVs) has become a common approach for the treatment of patients with symptomatic atrial fibrillation.^1^ The relative simplicity, fast learning curve, and lower interoperator variability have resulted in widespread adoption of this technique in clinical practice.^2^ Recently, a second cryoballoon technology (POLARx; Boston Scientific, Marlborough, MA) was introduced that seems to have a similar efficacy and safety as the fourth-generation Arctic Front Advance Pro (Medtronic, Minneapolis, MN).^3^, ^4^, ^5^, ^6^, ^7^ In general, during a cryoballoon procedure the transseptal access is gained through a standard 8F transseptal sheath with the use of a transseptal needle. After placement of the guidewire in the PV, the transseptal sheath is replaced by a larger steerable sheath (15.9F POLARSHEATH; Boston Scientific or 15F FlexCath; Medtronic) to accommodate the cryoballoon. Recently, the novel AcQCross Qx (Acutus Medical, Carlsbad, CA) system was introduced, which is an integrated needle and dilator that allows a 0.032” guidewire to be loaded during the transseptal puncture (Figure 1). This provides the ability to position, reposition, and cross the fossa ovalis without removing the guidewire. The AcQCross Qx family can be used in combination with many transseptal sheaths, including the 15.2F AcQGuide Max 2.0 steerable sheath (Acutus Medical). This crossing device streamlines the procedural workflow by eliminating the need for transseptal sheath exchange. We report the first case of the use of the AcQCross transseptal system during a POLARx cryoballoon procedure.

## Case report

A 55-year-old man was referred to our center for catheter ablation of symptomatic drug-refractory persistent atrial fibrillation requiring several electrical cardioversions. He had a normal left ventricular function and no left atrial (LA) dilatation. His CHA_2_DS_2_-VASc score was 0. We scheduled him for a PV isolation using a cryoballoon. His cardiac medication at admission included dabigatran and amiodarone. Dabigatran dose was withheld on the morning of the procedure. The procedure was performed under deep sedation by the anesthetist. After sedation, transesophageal echocardiography ruled out thrombus in the LA appendage. Ultrasound-guided venous access was gained in the right femoral vein with a shallow angle of entry, after which 2 sheaths (8F and 10F) were placed. Directly after femoral access an initial bolus of heparin (100 U/kg) was given and this bolus was repeated after transseptal puncture with start of a continuous heparin infusion to achieve an activated clotting time of 300 seconds. An intracardiac echocardiography catheter (ViewFlex Xtra, St. Jude Medical/Abbott, Lake Bluff, IL) was placed in the right atrium to visualize the fossa ovalis. The combination of AcQCross Qx integrated needle and dilator and 15.2F AcQGuide Max 2.0 transseptal sheath was placed in the superior vena cava over a 0.032” guidewire. The passage of the transseptal sheath at the femoral access was without resistance. Subsequently, the transseptal sheath was withdrawn from the superior vena cava to the fossa ovalis under guidance of intracardiac ultrasound and fluoroscopy. After transseptal puncture with the spring-tensioned hollow crossing needle of AcQCross Qx, the retained 0.032” guidewire was advanced to the left superior PV (Figure 1, Online Supplemental Videos 1 and 2). The steerable sheath was then inserted in the left atrium over the guidewire. After slow aspiration and flushing of the transseptal sheath with heparinized saline, a long-tip (12 mm) POLARx cryoballoon was inserted through the AcQGuide Max 2.0 transseptal sheath to the LA, without notable resistance. Fluoroscopy was needed to determine the position of the cryoballoon catheter relative to the tip of the steerable transseptal sheath. We used cryoablation applications of 180 seconds in duration if the time to isolation was 60 seconds or less. Otherwise a 240-second cryoablation application was performed. Right phrenic nerve pacing was performed during cryoablation of the right PVs to reduce risk of phrenic nerve injury. Phrenic nerve capture was monitored using a movement sensor (Diaphragm Movement Sensor; Boston Scientific). If there was a loss of phrenic nerve capture, the cryoablation was immediately stopped. Isolation of all 4 PVs was achieved after 5 cryoapplications. One cryoapplication (#3) in the right inferior PV was prematurely terminated at 71 seconds owing to a decrease in Diaphragm Movement Sensor (<65%), but this was secondary to dislocation of the quadripolar electrophysiology catheter used for pacing the phrenic nerve. Resheathing of the cryoballoon after each cryoapplication was done without notable resistance. At the end of the procedure, entry and exit block was confirmed in every PV. Closure of the femoral access site was achieved by using a purse-string suture. The total procedure time (from sheath insertion to removal) was 47 minutes; the LA dwelling time was 38 minutes; fluoroscopy time was 10 minutes; and ablation time was 851 seconds. The patient was discharged the following morning without in-hospital complications.

## Discussion

We present the first reported case of the use of a novel integrated dilator and needle system (AcQCross Qx) in combination with a 15.2F steerable sheath (AcQGuide Max 2.0) to perform a cryoballoon procedure with a POLARx cryoballoon catheter. This case highlights several important aspects of the availability of this approach for cryoballoon procedures.

The AcQCross Qx system provides a unique crossing system that eliminates the need for a separate transseptal needle. The curved dilator contains an integrated spring-tensioned hollow needle, which allows retention of a 0.032” guidewire through the entire transseptal procedure. The possibility of a retained guidewire results in easy repositioning and reduces exchanges. The retained guidewire permits direct placement of the guidewire in the left superior PV after transseptal puncture to confirm proper transseptal puncture. Thus, in case of inadvertent transseptal puncture only the needle is perforated. Considering the large-bore transseptal access, it is important to realize that the dilator and sheath are only advanced after confirmation of proper location of the guidewire. The dilator/needle provides excellent echogenicity at the fossa ovalis (Online Supplemental Video 2). Furthermore, the dilator can be reshaped to match the anatomy of the patient. If there is difficult transseptal access then there is also the ability to use radiofrequency at the needle to facilitate septal crossing.

Unfortunately, the use of a 0.035” (or larger) guidewire for more support is not possible, as it is not compatible with the current AcQCross Qx system. As the AcQCross Qx system consists of a large curved dilator, it is important that the femoral vein access be performed with a shallow angle of entry, and in case of resistance at the vein entry site predilation with a 14F short dilator is recommended. After predilation, a clockwise and counterclockwise movement of the dilator/sheath combination can ease crossing of the dilator/sheath at the vein entry site. In our case, we did not experience any resistance at the femoral access site.

**Figure 2:**
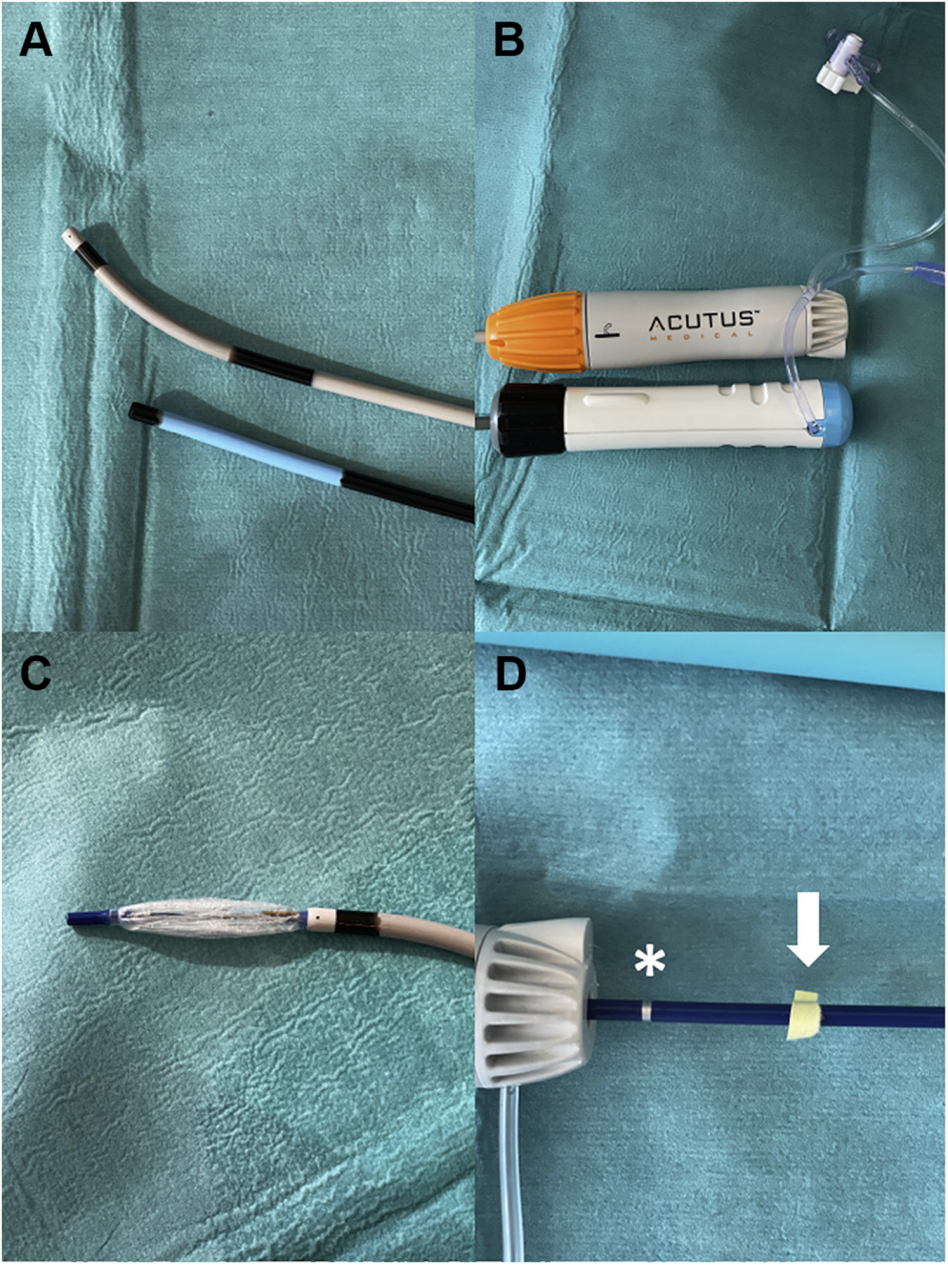
**A, B:** Differences in total length between the AcQGuide Max 2.0 sheath (Acutus Medical, Carlsbad, CA) and POLARSHEATH (Boston Scientific, Marlborough, MA). When the cryoballoon of the POLARx catheter (Boston Scientific) is just outside the AcQGuide Max 2.0 sheath (**C**), the proximal marker (∗) on the shaft of the POLARx catheter is approximately 2.5 cm too distal when used in the AcQGuide Max 2.0 sheath (**D**). When the yellow tape (*arrow*) is at the hub of the handle then the cryoballoon is just outside the sheath (position noted in **C**).

The AcQCross Qx dilator/needle family is compatible with different sheaths (length- and size-matched) and has a dedicated system for the FlexCath Advance steerable sheath (AcQCross Qx – FC 65 cm). Currently, there is no specific AcQCross Qx dilator/needle system compatible with the POLARSHEATH; therefore we used the AcQGuide Max 2.0 steerable sheath for our POLARx case. The inner and outer diameters of the POLARSHEATH (12.7F and 15.9F, respectively) are slightly larger than the AcQGuide Max 2.0 (12.4F and 15.2F, respectively). However, the slightly smaller inner lumen of the AcQGuide Max 2.0 sheath did not cause noticeable resistance while inserting or resheathing the POLARx cryoballoon catheter during the case. A potential advantage of the AcQGuide Max 2.0 sheath is that it can provides more tip deflection than the POLARSHEATH (180 vs 155 degrees of deflection). This may be useful when approaching the right inferior PV with a hockey stick maneuver. Furthermore, crossing of the femoral access point and fossa ovalis was relatively easy owing to the slim crossing profile of the sheath/dilator combination. A disadvantage of using the AcQGuide Max 2.0 is that the markers on the shaft of the POLARx cryoballoon catheter are not indicating the correct position of the cryoballoon in relationship to the tip of the steerable sheath. The distal marker on the shaft indicates that the POLARx cryoballoon catheter is just inside the POLARSHEATH, while the proximal marker on the shaft indicates that the cryoballoon is just outside the sheath. The overall length of AcQGuide Max 2.0 is longer (∼2.5 cm) than the POLARSHEATH; therefore the markers are too distal in relationship to the entry of the sheath (Figure 2). Fluoroscopy is necessary to determine whether the cryoballoon catheter is just inside the transseptal sheath or whether the balloon is just outside the sheath. As the latest version of the POLARx cryoballoon catheter does not have a proximal radiopaque marker, care must be taken to verify that the cryoballoon is fully out of the sheath before inflation.

Although we used the AcQGuide Max sheath, it is important to highlight that the use of a transseptal sheath from a different manufacturer than the cryoballoon has the inherent risk of incompatibility because this specific combination was not tested for use. Thus, in case of errors the cryoballoon manufacturer may not give support, including not replacing the defective cryoballoon catheter because it was not used with the dedicated sheath. It is likely that Acutus Medical will develop a dedicated AcQCross Qx system for the POLARSHEATH in the near future, which gives the opportunity to perform a POLARx case with a POLARSHEATH in combination with the AcQCross Qx system to mitigate abovementioned issues.

## Conclusion

In conclusion, we demonstrate the feasibility of performing a POLARx cryoballoon procedure with the combination of the AcQCross Qx system with the AcQGuide Max 2.0 steerable sheath. The elimination of the need for transseptal sheath exchange improves the procedural workflow. Theoretically, reduction of sheath exchanges may also improve safety. Larger case series are warranted to confirm whether this novel approach for gaining transseptal access enhances procedural efficacy during cryoballoon procedures.

## Appendix Supplementary data

Supplemental Video 1 Cine film (left anterior oblique view) during transseptal puncture with the AcQCross Qx system in the AcQGuide Max 2.0 sheath. Please note puncture with the needle followed by placement of the 0.032” guidewire in the left superior pulmonary vein. The ViewFlex ICE catheter is positioned in the right atrium to visualize the fossa ovalis as shown in video 2.

Supplemental Video 2 Intracardiac echocardiographic imaging during transseptal puncture with the AcQCross Qx system in the AcQGuide Max 2.0 sheath. There is tenting of the fossa ovalis, followed by transseptal puncture with the needle and crossing of the 0.032” guidewire to the left atrium. Please note the high echogenicity of the needle during the puncture.

Supplementary material file ▪▪▪
