# Supplementary material for: Optimization of the transseptal procedural workflow using a novel integrated dilator and needle during a cryoballoon procedure
Source: HeartRhythm Case Rep. 2021 Nov 9;8(1):60–3. doi: 10.1016/j.hrcr.2021.11.001 (PMC8767177; doi:10.1016/j.hrcr.2021.11.001)
Supplement: Supplementary material file — ▪▪▪ [file mmc3.docx]

**Legends supplementary videos**

Video 1. Cine film (left anterior oblique view) during transseptal puncture with the AcQCross Qx system in the AcQGuide Max 2.0 sheath. Please note puncture with the needle followed by placement of the 0.032” guidewire in the left superior pulmonary vein. The ViewFlex ICE catheter is positioned in the right atrium to visualize the fossa ovalis as shown in video 2.

Video 2. Intracardiac echocardiographic imaging during transseptal puncture with the AcQCross Qx system in the AcQGuide Max 2.0 sheath. There is tenting of the fossa ovalis, followed by transseptal puncture with the needle and crossing of the 0.032” guidewire to the left atrium. Please note the high echogenicity of the needle during the puncture.
